# Not Only Systemin: Prosystemin Harbors Other Active Regions Able to Protect Tomato Plants

**DOI:** 10.3389/fpls.2022.887674

**Published:** 2022-05-24

**Authors:** Donata Molisso, Mariangela Coppola, Martina Buonanno, Ilaria Di Lelio, Anna Maria Aprile, Emma Langella, Maria Manuela Rigano, Silvana Francesca, Pasquale Chiaiese, Gianna Palmieri, Rosarita Tatè, Martina Sinno, Eleonora Barra, Andrea Becchimanzi, Simona Maria Monti, Francesco Pennacchio, Rosa Rao

**Affiliations:** ^1^Department of Agricultural Sciences, University of Naples Federico II, Naples, Italy; ^2^Institute of Biostructures and Bioimaging, National Research Council (IBB-CNR), Naples, Italy; ^3^Institute of Biosciences and BioResources, National Research Council (IBBR-CNR), Naples, Italy; ^4^Institute of Genetics and Biophysics, National Research Council (IGB-CNR), Naples, Italy; ^5^Interuniversity Center for Studies on Bioinspired Agro-Environmental Technology (BAT Center), University of Naples Federico II, Naples, Italy

**Keywords:** fragments, natively unfolded, bioactivity, tomato protection, insect herbivores, phytopathogenic fungi, endogenous defense, not direct toxicity effect

## Abstract

Prosystemin is a 200-amino acid precursor expressed in Solanaceae plants which releases at the C-terminal part a peptidic hormone called Systemin in response to wounding and herbivore attack. We recently showed that Prosystemin is not only a mere scaffold of Systemin but, even when deprived of Systemin, is biologically active. These results, combined with recent discoveries that Prosystemin is an intrinsically disordered protein containing disordered regions within its sequence, prompted us to investigate the N-terminal portions of the precursor, which contribute to the greatest disorder within the sequence. To this aim, PS1-70 and PS1-120 were designed, produced, and structurally and functionally characterized. Both the fragments, which maintained their intrinsic disorder, were able to induce defense-related genes and to protect tomato plants against *Botrytis cinerea* and *Spodoptera littoralis* larvae. Intriguingly, the biological activity of each of the two N-terminal fragments and of Systemin is similar but not quite the same and does not show any toxicity on experimental non-targets considered. These regions account for different anti-stress activities conferred to tomato plants by their overexpression. The two N-terminal fragments identified in this study may represent new promising tools for sustainable crop protection.

## Introduction

Plants are constantly challenged by different pests and pathogens that negatively affect crop performance and yield, imposing severe economic losses. Chemical pesticides still represent a crucial and important tool for pest control in agriculture, which generates a wealth of problems, given their negative impact on ecosystem service providers, environment and human health (Bernardes et al., 2015; Tudi et al., 2021). Public awareness of such problems stimulated an increasing demand of safe food products, with low or no pesticide residues, obtained using ecologically sustainable production management (Zikankuba et al., 2019; Gomes et al., 2020). To further promote this process, and, more generally, a one-health vision underlying a sustainable management strategy of our planet, the United Nations declared 2020 as the International Year of Plant Health^1^.

Considerable efforts are currently being devoted to the development of new tools and strategies for sustainable pest control. A special attention has been given to the use of natural antagonism and of regulating molecules and genes that mediate pest suppression mechanisms shaped by long-term co-evolutionary processes, driven by the continuous contrast between immune defense barriers and virulence strategies (Gupta and Dikshit, 2010; Pennacchio et al., 2012; Coppola et al., 2019; Hay et al., 2020; Basaid et al., 2021). This approach represents a further development of biological control which goes beyond the organism level and exploits all types of natural antagonism.

In this context, a wealth of information on plant-pest/pathogen interactions is becoming increasingly available, offering new opportunities for plant protection. The first line of inducible plant defense is triggered by the detection of non-self molecules which are called pathogen/herbivore-associated molecular patterns (PAMPs, HAMPs). PAMPs and HAMPs activate the so-called pattern-triggered immunity (PTI) (Bigeard et al., 2015; Abdul Malik et al., 2020). Plant perception of these highly conserved molecules is mediated by plasma membrane-localized pattern recognition receptors (PRRs), that initiate the PRRs-mediated immune responses triggering a complex cascade of signaling events including depolarization of cellular membrane, rapid transient fluctuations in the concentration of intracellular calcium ions, production of reactive oxygen species (ROS), activation of mitogen-activated protein kinases (MAPKs) and extensive transcriptomic and metabolomic reprogramming (Bigeard et al., 2015; Trapet et al., 2015). The amplification of immune signals rapidly after the PAMPs/HAMPs perception is a key-event to efficiently counteract pest invasion and it is mediated by plant sensitivity to some host derived molecules, named damage-associated molecular patterns (DAMPs), and partially overlapping PTI signaling components (Lotze et al., 2007; Boller and Felix, 2009). DAMPs include mainly cell wall protein or polysaccharide fragments, peptides, amino acids, and nucleotides (Boller and Felix, 2009; Henry et al., 2012; Thakur and Sohal, 2013) released from the damaged tissues following pest invasion. Exogenous treatment of plants with DAMPs activates a wide range of defense responses, including the production of ROS and the expression of defense-related genes (Hou et al., 2019). A well characterized class of plant DAMPs are peptides released from precursor proteins (Choi and Klessig, 2016). These include a family of small defense-related peptide hormones called systemins, that are released from larger precursors by plants of *Solanaceae* family in response to wounding and herbivore attack (Pearce et al., 1991; Ryan and Pearce, 2003).

Tomato Systemin (Sys) is a small peptide consisting of 18 residues released from the C-terminal region of a 200 amino acid precursor protein called Prosystemin (ProSys) (Beloshistov et al., 2018). *ProSys* gene is transcribed at very low level in unchallenged plants, while its expression greatly increases in response to mechanical wounding or insect herbivore attack (McGurl et al., 1994; Ryan, 2000). The role of ProSys in plant defense against wounding and biotic damages was clearly demonstrated through the analysis of transgenic tomato plants expressing the *ProSys* gene. Indeed, the constitutive production of ProSys resulted in an increased plant resistance to several biotic stress agents due to a wide transcriptomic reprogramming that included the up-regulation of an array of defense-related genes involved in both direct and indirect defenses (McGurl et al., 1994; Ryan, 2000; Dıaz et al., 2002; Corrado et al., 2007; Degenhardt et al., 2010; El Oirdi et al., 2011; Coppola et al., 2015). In addition, it was observed that transgenic plants tolerate moderate salinity stress (Orsini et al., 2010), virus spread (Bubici et al., 2017) and, more recent evidence indicate a reduction of nematode colonization (Martínez-Medina et al., 2021).

The mechanisms that underpin the wide array of activated defense barriers are not fully understood and appear to be in part linked to the structure of the protein precursor. A detailed biophysical and biochemical characterization of ProSys revealed that the precursor is a member of the Intrinsically Disordered Protein (IDP) family (Buonanno et al., 2018), containing Intrinsically Disordered Regions (IDRs) within its sequence. IDPs are highly flexible proteins lacking a stable or ordered three-dimensional structure. It has been demonstrated that IDPs have crucial roles in cellular processes by conferring functional advantages in stress response, signaling and regulation (Sun et al., 2013; Uversky, 2013). The structural flexibility allows them to assume alternative conformations which, according to specific conditions, prompt their transient, but specific, interactions with multiple molecular partners (Uversky, 2013; Wright and Dyson, 2015; Covarrubias et al., 2017). It is then possible that different regions of the precursor interact with different partners activating concurrent defense signals. Consistent with this, we previously demonstrated that the expression in tobacco plants of a truncated ProSys gene, lacking the exon encoding Sys, altered the proteomic profile and increased plant resistance against *Botrytis cinerea* (Corrado et al., 2016). Recently, effects on tomato defense of either transgenic tomato plants expressing a truncated ProSys gene or tomato plants treated with recombinant truncated Prosys_(1–178)_ (devoided of Sys sequence) were investigated (Molisso et al., 2022). In both cases it was observed a clear modulation of the expression of genes linked with defense responses which resulted in protection against the lepidopteran pest *Spodoptera littoralis* and the fungus *Botrytis cinerea*. In order to further identify the regions of ProSys scaffold which confer biological activity, we designed and produced two different recombinant ProSys fragments starting from the N-terminal part of the protein, namely PS1-70 and PS 1-120, which contributed to the greatest content of disorder. These two fragments were structurally and functionally characterized. We demonstrated that both fragments, which maintained the intrinsic disorder of the precursor, induce defense-related genes and protect tomato plants against *Botrytis cinerea* and *Spodoptera littoralis* larvae. Intriguingly, the biological activity of each of the two fragments and of Sys was similar but not quite the same with each other and with the Sys peptide used as comparison. We also monitored the direct toxic effect of these two fragments against two different cellular cultures: a photosynthetic microorganism *Chlamydomonas reinhardtii* and a human keratinocyte HaCaT cells line. As expected PS1-70 and PS1-120 did not show any toxicity on experimental non-targets considered making them novel and interesting tools to be used for crop protection. Taken together, our results confirm previous hypothesis that ProSys is not only a mere scaffold of Sys but contains multiple biologically active sequences, different from the well-known Sys peptide, which have been here identified and characterized.

## Materials and Methods

### Plant Material and Growth Condition

Tomato seeds (*Solanum lycopersicum* L. cultivar “Dwarf San Marzano”) were surface sterilized with 70% ethanol for 2 min, rinsed, washed with 2% sodium hypochlorite for 10 min and rinsed five times with sterile distilled water. Seeds were germinated in Petri dishes on wet sterile paper and placed in a growth chamber at 24 ± 1°C and 60 ± 5% of relative humidity (RH) in darkness. Upon roots emergence, plantlets were transferred to a polystyrene plateau with barren substrate S-type (Floragard, Oldenburg, Germany) in a growth chamber at 26 ± 1°C and 60 ± 5% RH, with 18:6 h light/dark photoperiod, at a brightness of 5,000 lux. After 2 weeks, plants were transferred to sterile soil mixture in pots of diameter of 9 cm and grown using the same growth conditions.

### Molecular Cloning, Expression, and Purification of ProSys Fragments

Two DNA fragments, PS1-70 and PS1-120, were amplified from the full-length ProSys cDNA, using the primers reported in Supplementary Table 1 and as described by Buonanno et al. (2018); PCR-fragments were cloned in pETM11 vector (a kind gift from EMBL, Heidelberg) using *Nco*I- *Xho*I restriction sites. The generated plasmids were checked by appropriate digestion with restriction enzymes and nucleotide sequencing. Optimized expression of the recombinant ProSys fragments was obtained in *E. coli* BL21(DE3) cells in different selective media: LB (Luria-Bertani) for PS1-70 and 2-YT (Yeast extract-Tryptone) for PS1-120. Large-scale production was carried out inducing with 2 mM IPTG (Isopropyl β-D-1-thiogalactopyranoside) for 16 h at 22°C. Cells were harvested by centrifugation (20 min at 6,000 *g* at 4°C) and resuspended in lysis buffer [20 mM Tris–HCl, 20 mM imidazole, 50 mM NaCl, 1 mM dithiothreitol (DTT), pH 8.0] in presence of 0.1 mM phenylmethanesulfonylfluoride (PMSF), 5 μg/ml DNaseI, 0.1 mg/ml lysozyme and 1X protease inhibitors (Sigma-Aldrich, Milan, Italy). Cells were disrupted by sonication on ice and after centrifugation (30 min at 30,000 *g* at 4°C) the supernatant of each ProSys fragment was purified by an ÄKTA FPLC, on a 1 ml HisTrap FF column (GE Healthcare, Milan, Italy), according to manufacturer’s instruction (GE Healthcare Milan, Italy). After elution, ProSys fragments were dialyzed in 20 mM Tris–HCl, 50 mM NaCl, 100 mM PMSF, 1 mM DTT, pH 8.0 and purified by size exclusion chromatography (SEC) on a Superdex 75 10/300 HP (GE Healthcare Milan, Italy), in PBS 1X (Phosphate buffer saline, 10 mM phosphates, 140 mM NaCl, 2.7 mM KCl, pH 7.4, Sigma-Aldrich St. Louis, MO, United States), 100 μM PMSF, 1 mM DTT pH 8.0. Calibration was carried out using the following standards (Sigma Aldrich, St. Louis, MO, United States): horse cytochrome c (Cit c, 12.4 kDa), chicken ovalbumin (Ova, 45 kDa), bovine serum albumin (BSA 66 kDa), carbonic anhydrase from bovine erythrocytes (CA, 29 kDa), recombinant carbonic anhydrase XIV (CA XIV, 37 kDa, homemade) and the full-length Prosystemin (ProSys, 26 kDa, homemade). The purity level of the recombinant fragments was assessed by SDS-PAGE on a 15% gel using Biorad Precision Plus Protein All Blue Standards (10–250 kDa) as molecular mass marker. LC-ESI-MS analysis of the protein, performed as previously described (Buonanno et al., 2018; Langella et al., 2018) confirmed their identities.

### Light-Scattering Analysis

Light scattering analysis of PS1-70 and PS1-120 was performed as previously described in Alterio et al. (2019) by combining SEC with MALS-QELS (MultiAngle Ligh Scattering-Quasi-Elastic Light Scattering) detectors equipment. Experiments were run at 0.5 ml/min PBS 1X, 100 μM PMSF, 1 mM DTT, pH 8.0 on a Biosep-SEC-s2000 column (Phenomenex, Torrance, CA, United States) linked to an ÄKTA FPLC coupled to a light scattering detector (mini-DAWN TREO, Wyatt Technology) and to a refractive index detector (Shodex RI-101). All data collected were processed using the ASTRA 5.3.4.14 software (Wyatt Technologies Corporation).

### Circular Dichroism Spectroscopy

Measurements were performed on a Jasco J-715 (Easton, MD, United States) spectropolarimeter equipped with a Peltier temperature control system (Model PTC-423-S), using a Hellma quartz cell of 0.1-cm-path length in the far-UV from 190 to 260 nm (20 nm/min scan speed). Circular dichroism (CD) spectra, collected as previously described (D’Agostino et al., 2019) were signal averaged over at least three scans, and the baseline was corrected by subtracting the buffer spectrum. Spectra were recorded at 20°C in 10 mM sodium phosphate buffer pH 7.4 at fragments concentrations for PS1-70 and PS1-120 of 4.4 and 3.5 μM, respectively. The same parameters were applied for measurements in the temperature range of 10–80°C. Spectra were collected every 10°C. Molar ellipticity values, recorded at 222 nm for both experiments were plotted as function of the temperature using GraphPad Prism version 6.01 (GraphPad software; San Diego, California, United States).

Titration with increasing concentration of trifluoroethanol (from 0% up to 25%TFE), measurements were performed at 20°C for fragment concentration of 5.15 and 3.5 μM, for PS1-70 and PS1-120, respectively. DICHROWEB^2^ (Whitmore and Wallace, 2004) was used to analyze data. CDSSTR was used as a deconvolution method (Whitmore and Wallace, 2008) to evaluate the percentage of α-helical content of the ProSys fragments.

### Sequence Analysis

The primary sequence of PS1-70 and PS1-120 was analyzed using Composition Profiler tool^3^ (Vacic et al., 2007). The query samples were compared with the reference value of the average amino acid frequencies of the Swiss-Prot database^4^ (Vacic et al., 2007). Composition analysis was carried out using the relation (CPX –CSX)/CSX, where CPX means the content of an amino acid X within the protein of interest, whereas CSX is the typical composition of X in Swiss-Prot proteins. In the final output, the less abundant amino acids are represented with negative values, whereas those more abundant with positive values.

### Plant Treatments and Gene Expression Analysis

Healthy and fully expanded leaves of four-week-old plants were treated with 100 fM purified recombinant fragments. In particular, 15 spots of 2 μl of 100 fM PS1-70 or PS1-120 were gently placed on the adaxial surface of the intact leaves. A similar treatment was done with Sys obtained as previously described (Coppola et al., 2019), while a mock treatment with PBS was used as control. Leaf samples were collected 6 hpt for bioassays and molecular analyses, unless otherwise indicated.

### Bioassays

#### *Spodoptera littoralis* Bioassay

*Spodoptera littoralis* larvae (Lepidoptera, Noctuidae) were reared on artificial diet at 25 ± 1°C and 70 ± 5% RH, with 16:8 h light-dark photoperiod as previously described (Di Lelio et al., 2014). Newborn larvae were allowed to grow on this artificial diet until they attained the 2^nd^ instar. Then, newly molted 3^rd^ instar larvae were selected, weighted and singly isolated in 4-wells plastic rearing trays (RT32W, Frontier Agricultural Sciences, Pitman, NJ, United States). In each well, 3 ml of 1.5% agar-agar (w/v) were dispensed, in order to keep tomato leaves turgid, in a moist environment, and the rearing wells were closed with perforated plastic lids (RTCV4, Frontier Agricultural Sciences, Pitman, NJ, United State). In order to select the most appropriate concentration of Sys, PS1-70 and PS1-120 on the survival rate of larvae alimented with treated leaves we compared 100 pM and 100 fM concentrations in a preliminary assay. For each treatment, 32 larvae were fed with leaf disks of tomato plant. The experimental larvae were maintained at the rearing condition described above. Larvae were weighted every 2 days and mortality was daily checked until pupation.

#### Larval Toxicity Assay

The larvicidal assays were carried out on 4^th^ instars, following topical exposure or oral ingestion, as reported elsewhere (Pavela, 2010), using a range of three experimental doses (100 nM, 100 pM and 100 fM); for each dose, 16 *S. littoralis* larvae were treated. Briefly, for topical application the larvae were anesthetized on ice an directly treated with the solution containing the ProSys fragments through the direct application of 1 μl of the solution containing the ProSys fragments on the neck membrane, using a Gilson pipette P10 (PIPETMAN classic P10), while for oral ingestion 1 μl of experimental solutions was poured into the foregut lumen of the anesthetized larvae by means of a Hamilton Microliter syringe (1701RNR 10 ll, gauge 26s, length 55 mm, needle 3). Control larvae were identically treated with PBS 1X buffer.

After the treatments, the larvae were singly separated into polystyrene rearing trays, provided with food and kept at the rearing conditions reported above. Larval mortality was recorded until pupation, which took place into plastic boxes containing vermiculite (25 cm × 10 cm × 15 cm).

#### *Botrytis cinerea* Bioassay

*Botrytis cinerea* spores were cultivated on MEP (Mannitol Egg yolk Polymyxin) solid medium at 22°C. Spores were collected by washing the agar surface with sterile distilled water containing 0.1% Tween 20, filtered through sterile Kimwipes (Kimberly-Clark Dallas, Texas, United States), to remove fragments of hyphae, and adjusted to a concentration of 1 × 10^6^ conidia/ml. Ten μl drops of spore suspension were put on tomato leaves, 6 hpt with 100 fM ProSys fragments, using 10 different inoculation points per detached leaf. The assay was carried out using for each compound leaves from 5 different plants. Detached leaves were placed on sponges soaked in sterile water and incubated in a growth chamber at 23°C, under 16:8 h light/dark photoperiod and 90% ± 5% RH. The size of the lesions was measured at 1, 3, 5, and 8 days after infection using a digital caliper.

#### *In vitro* Antifungal Assay

The antifungal assay was carried out as already reported (Pastor-Fernández et al., 2020; Molisso et al., 2021). A sterile 12-well plate was filled with potato dextrose broth (PDB 1/2) medium, containing ProSys fragments at the final concentration of 100 fM. A solution with *B. cinerea* spores was added to each well, in order to reach a final concentration of 10^4^ spores/ml, then the plate was placed in a shaker and incubated for 24 h at 25 ± 1°C. To assess the fungal growth, the value of optical density (OD) at a wavelength of 600 nm (OD_600_) was measured in triplicate on a BioPhotometer Spectrophotometer UV/VIS (Eppendorf, Hamburg, Germany).

### Gene Expression Analysis

Total RNA extraction, synthesis of the first strand cDNA and Real Time-PCRs (RT-PCR) were carried out according to standard procedure, as already published (Corrado et al., 2012). RT-PCR was performed using Rotor Gene 6000 (Corbett Research; Sydney, Australia). Gene expression analysis was carried out using two technical replicates for each of the three biological replicates per samples. The housekeeping *EF-1*α gene was the endogenous reference gene used for the normalization of the expression levels of the target genes (Marum et al., 2012; Müller et al., 2015). Relative quantification of gene expression was carried out using the 2^–ΔΔCt^ method (Livak and Schmittgen, 2001). Primers and their main features are described in Supplementary Table 2.

### Hydrogen Peroxide and Malondialdehyde Determination

Quantification of H_2_O_2_ content was carried out by using a colorimetric method (Sergiev et al., 1997). Briefly, 0.1 g of frozen powder from tomato leaves were extracted with 1 ml of ice-cold 0.1% trichloroacetic acid (TCA) and the mixture was then incubated for 15 min on ice and centrifuged at 10,000 rpm for 15 min at 4°C. Subsequently, 500 μl phosphate buffer (pH 7.0) and 1 ml of potassium iodide 1 M were added to 500 μl of supernatant. The mixture was then incubated in the dark for 40 min and measured at 525 nm, using a Nano Photometer TM (Implen, Munich, Germany). Three separate biological replicates for each sample and three technical assays for each biological replicate were measured. The concentration was expressed in mmol^–1^g FW (Fresh Weight).

The malondialdehyde (MDA) levels in leaf tissues indicate the levels of membrane lipid peroxidation. For the determination of MDA, 0.2 g of leaf sample was ground after adding 1 ml of ice cold 0.1% trichloroacetic acid (TCA). The samples were incubated for 15 min on ice and centrifuged at 10,000 rpm for 10 min at 4°C. Subsequently, 250 μl of the supernatant was mixed with 1,250 μl reaction solution (TCA 20% + 2-thiobarbituric acid (TBA) 0.5%), water-bathed for 30 min at 95°C and measured at 532 and 600 nm using a Nano Photometer TM (Implen, Munich, Germany). Three separated biological replicates for each sample and three technical assays for each biological replicate were measured. The concentration was expressed as quantity of MDA-TBA complex (Zhang and Kirkham, 1996).

### Toxicity Bioassays of Protein Fragments on Microalgae and on Human Keratinocyte

Cultures of *Chlamydomonas reinhardtii* were axenically grown under phototrophic conditions, in a mineral nutrient solution (MNS) at 24 ± 1°C, with a rotatory agitation at 100 rpm with a 16:8 h light/dark photoperiod, at 100 E m^–2^ s^–1^ (Bischoff, 1963; Chiaiese et al., 2011). The cultures were weekly maintained by inoculation of 500 μl of culture in 50 ml of MNS medium.

The growth inhibition assay was assessed following (Lewis, 1994) and (OECD, 2011) procedures with some modification. A replicated design was applied by exposing microalgae at three different concentrations of ProSys fragments; 0.0001, 0.1 and 1 nM in 24 well plate and incubated for 7 days. Microalgae incubated in the growth medium without ProSys fragments were used as control. Cell density was determined every 24 h by direct counting under a Leica DMR optical microscope (Leica Imaging Systems, Cambridge, United Kingdom).

The growth rates (μ) and percentages of growth inhibition (%I) at 96 h were calculated using the following equations:


$$\mu =\frac{l\,n\,N_{2}-l\,n\,N_{1}}{t_{2}-t_{1}}$$

where N_2_ and N_1_ are the cell density at times t_2_ and t_1_


$$\%I=\frac{\mu_{c}-\mu_{t}}{\mu_{c}}$$

where c is the specific growth rate in the control group and μ_*t*_ is the specific growth rate in each treatment. The data were expressed as means and standard deviation for three replicate cultures.

### Cell Culture and Maintenance

The human keratinocyte HaCaT cells line was obtained from the American type culture collection (CRC 1424; Virginia, MD, United States). HaCaT cells were incubated in Dulbecco’s modified eagle’s medium containing 10% heat-inactivated fetal calf serum, 1,000 U/ml penicillin and 100 mg/ml streptomycin at 37°C in a humidified atmosphere incubator containing 5% CO_2_.

### Cell Viability and Morphology Studies

To assess cell viability, cell counting Kit-8 (CCK-8 assay, ab228554; Abcam, Cambridge, MA, United States) assay and manual cell count were performed. The CCK-8 was a sensitive colorimetric assay for quantization of viable cell number in proliferation and cytotoxicity assays. The experiments were performed in cells by using the protocols recommended by the manufacturer. In brief, 70% confluent cultured flasks, containing 5 × 10^3^ cells/100 μl, were plated at 2 × 10^4^/ml per well into 24-well and the plates were incubated overnight at 37°C to allow cells to adhere to the bottom of wells. Then, cells were washed with PBS and exposed to PS-70 or PS1-120 fragments. Specifically, the ProSys fragments were suspended in DMEM medium and added to the cells at a final concentration of 100 nM. Non-treated cells were included as negative controls. After 24 h of incubation, Kit-8 reagent was added to each well, and the plate was further incubated at 37°C for 3 h. The absorbance at 450 nm was then recorded by a microplate reader. The cell viability was determined as follows: cell viability (%) = [(OD_experiment_ – OD_blank_)/(OD_negative control_ – OD_blank_)] × 100%, where OD_experiment_ is the absorbance of a well with a treated cell and CCK-8; OD_blank_ is the absorbance of a well with medium and CCK-8 but without cells; and OD_negative_ control is the absorbance of a well with untreated cells and CCK-8. For manual counts 50 μl of sample was mixed with 50 μl of 0.4% trypan blue by gently pipetting, and then 20 μl of the mix were loaded into each chamber of the hemocytometer. Counts were performed in triplicate by one analyst under a 20 × objective according to the standard methodology.

Afterward, the cells, untreated or treated with each fragment, were washed, fixed with paraformaldeide 4% and visualized by phase-contrast microscopy, using the DMI6000B inverted fully automated microscope with DFC 420 RGB camera (Leica Microsystems, Wetzlar, Germany). Leica LAS V5.4 software was utilized for image acquisition/elaboration (contrast/gamma adjusting). All analyses were performed in triplicate on three independent *in vitro* experiments.

### Statistical Analysis

Differences in survival rate were compared by using Kaplan–Meier and log-rank analysis.

One-Way ANOVA test, followed by the Tukey’s *post hoc* multiple comparison test (*P* < 0.05), was used to evaluate: the differences in larval weights and in necrosis diameter development, the effect of ProSys fragments on *B. cinerea* growth and infection, the differences in relative quantities of transcripts abundance, and the quantification of the amount of H_2_O_2_ and MDA levels. For the evaluation of ProSys fragments effect on microalgal growth, the statistical analysis was performed by One Way ANOVA coupled with Dunnet test. HaCaT cell viability assay was performed in triplicate on three independent sets of experiments. OD_450_ values were compared by using Student’s *t*-test (*P* < 0.05). Data were analyzed using GraphPad Prism version 6.01 (GraphPad software; San Diego, California, United States).

## Results

### Design, Production, and Characterization of PS1-70 and PS1-120 Fragments

**FIGURE 1:**
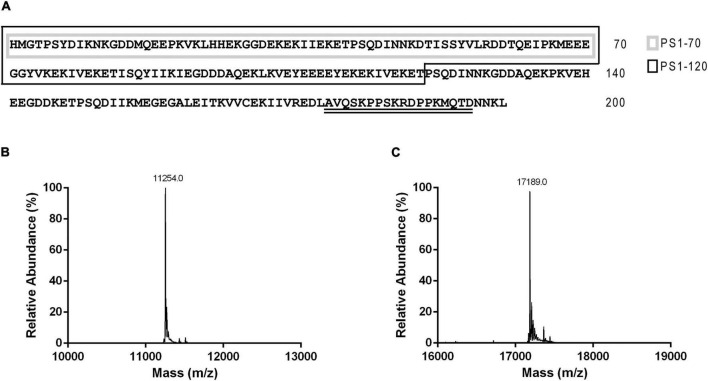
Primary sequences of ProSys and its fragments PS1-70 and PS1-120 and LC-ESI-MS analysis of the two fragments. **(A)** ProSys primary sequence: PS1-70 and PS1-120 sequences are indicated by a gray and a black box, respectively; Systemin sequence is double underlined. Deconvoluted mass spectrum of purified **(B)** PS1-70 and **(C)** PS1-120.

Based on the structural characterization of ProSys (Buonanno et al., 2018), we produced two fragments designed from the N-terminal part of the protein (Figure 1A), hereafter referred as PS1-70 and PS1-120, which were expressed and purified. PS1-70 and PS1-120 were designed based on our previous bioinformatic analyses carried out on the full-length ProSys protein by means of structure and disorder predictions (Buonanno et al., 2018). According to this data, ProSys was defined as an IDP, being characterized by the presence of both structured and long disordered regions. In details, PS1-70, which encompasses the first 70 residues starting from the N-terminal portion of the precursor, was predicted to be the most disordered fragment; on the contrary, PS1-120 which included 50 more residues encompassing the central part of the protein, was predicted to contain some secondary structure elements (Figure 1A). The rationale behind the design of the two constructs aimed to disclose potential structure-function relationships for these protein regions, which did not contain Sys peptide to avoid any overlapping biological effect with confounding effects.

Highly purified PS1-70 and PS1-120 were obtained following a two-step procedure with a final yield of about 2 mg/l of growth medium. The identity of the purified fragments (inclusive of the His-tag) was confirmed by LC-ESI-MS (Figures 1B,C). Similarly to ProSys, the recombinant fragments eluted from the SEC column with an anomalous retention volume, indicative of an apparent molecular mass (MM_app_) of 28.4 and 48.0 kDa, respectively, as estimated by the calibration curve (Supplementary Figure 1). Light scattering experiments confirmed that PS1-70 and PS1-120 are present in solution as monomers of about 9.36 ± 0.60 and 19.98 ± 1.50 g/mol, respectively, in full agreement with the MM_theo_ (Supplementary Figure 2) and with the solution behavior of ProSys. Indeed, the high presence of disorder-promoting residues, compared to the order-promoting ones (Supplementary Figure 3; Dunker et al., 2001; Radivojac et al., 2007; Midic and Obradovic, 2012; Uversky et al., 2013), conferred disordered features to PS1-70 and PS1-120.

### Conformational Features of PS1-70 and PS1-120 and Temperature Effects

**FIGURE 2:**
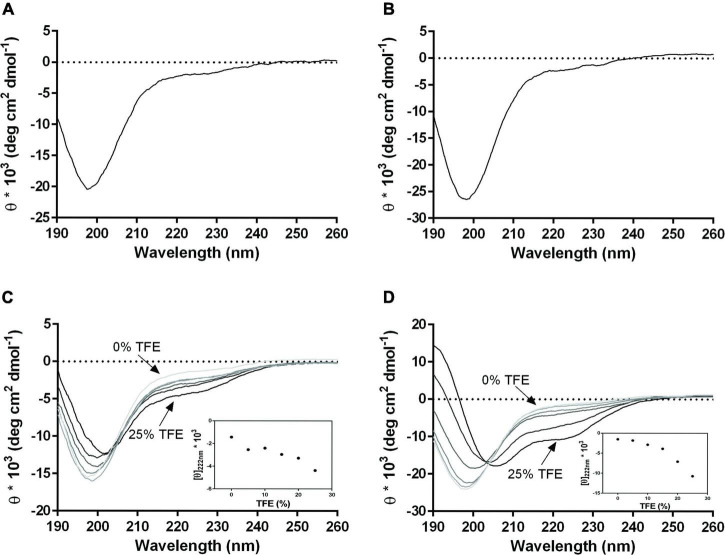
CD spectra of PS1-70 and PS1-120 and TFE titration. Far-UV CD spectra of **(A)** PS1-70 and **(B)** PS1-120. CD spectra overlapping of **(C)** PS1-70 and **(D)** PS1-120 recorded at increasing concentrations of TFE (0, 5, 10, 15 20, and 25%). Changes in molar ellipticity at 222 nm, as a function of TFE percentage are shown in the inset for both fragments. Spectra were acquired at 20°C in 10 mM phosphate buffer, pH 7.4.

The secondary structure of PS1-70 and PS1-120 was investigated by means of far-UV CD spectroscopy which measures the differential absorption of left- and right-circularly polarized light by chiral molecules. At these wavelengths the peptide bond contributions dominate, and the secondary structure give rise to a characteristic shape and magnitude of CD spectrum (Sreerama and Woody, 2004). In particular, a CD spectrum with a low ellipticity at 190 nm and a large negative ellipticity at 198 nm (Figures 2A,B) is typical of disordered proteins, confirming that, in agreement with previous investigations (Buonanno et al., 2018), also these two fragments have largely disordered conformation. The propensity of PS1-70 and PS1-120 to fold in water/trifluoroethanol (TFE) mixtures was evaluated, recording CD spectra at increased concentration of the co-solvent. The fragments behaved differently in presence of TFE. PS1-70 showed small differences in the CD spectra (Figure 2C), whereas PS1-120 displayed an increased α-helical content, as indicated by the positive maximum at near 190 nm and negative minima at 208 and 222 nm (Figure 2D).

Finally, PS1-70 and PS1-120 underwent modest temperature-induced changes, as already observed for the precursor. In fact, as the temperature increased to 80°C, only a small gain in secondary structures was observed (Supplementary Figure 4).

### Impact of PS1-70 and PS1-120 on Pests

**FIGURE 3:**
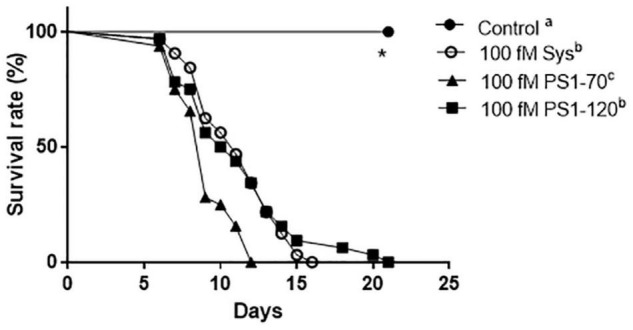
Effect of 100 fM Sys, PS1-70 and PS1-120 on *S. littoralis* larvae. Survival rate of experimental *S. littoralis* larvae. Different letters denote significant differences in the survival curve (Log-Rank test, *P* < 0.0001).

The results of the preliminary assay indicated that the most effective concentration of Sys, PS1-70 and PS1-120 in reducing larvae survival rate was the lowest (100 fM) (Supplementary Figure 5). Consequently, we used this concentration for all the other experiments. Supplementary Table 3 and Figure 3 showed the results observed for larvae fed with leaves treated with 100 fM Sys, PS1-70 and PS1-120: a very strong reduction of their weight and survival rates was registered in comparison with controls. The three experimental protein fragments showed a significant impact on larval survival [Log-rank (Mantel-Cox) test: χ^2^ = 110.8, dF = 3, *P* < 0.0001)], with PS1-70 treated leaf disks exerting a significantly stronger negative effect than leaves treated with Sys (Log-rank test: χ^2^ = 9.593, df = 1, *P* < 0.002) or with PS1-120 (Log-rank test: χ^2^ = 13.18, df = 1, *P* = 0.0003), which did not differ between them (Log-rank test: χ^2^ = 144, df = 1, *P* = 0.7356) (Figure 3).

No direct toxicity effect of PS1-70, PS1-120 and Sys on 4th larval instar of *S. littoralis* was observed as indicated in Supplementary Table 4. Both topical and oral applications, at different concentrations, had no larvicidal effect; all experimental larvae exhibited similar developmental patterns.

**FIGURE 4:**
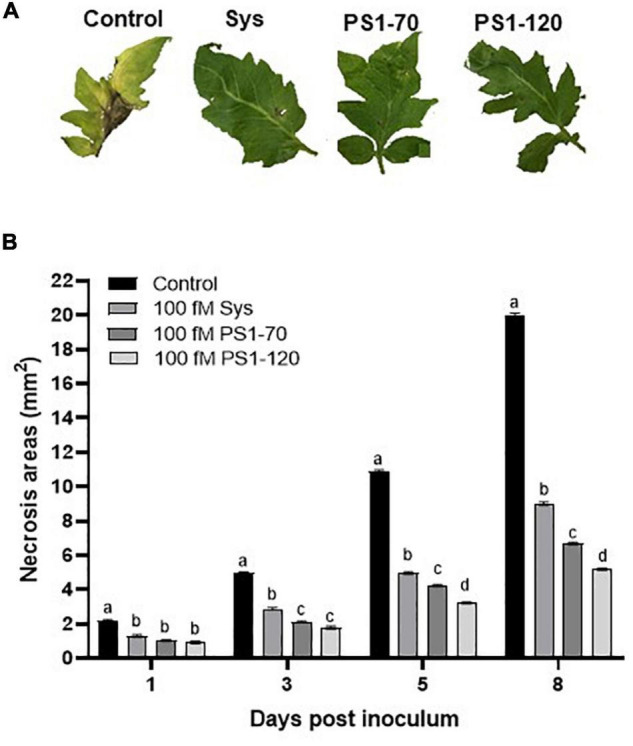
Enhanced resistance to *B. cinerea* by plants treated with ProSys fragments and Sys peptide. Response to *B. cinerea* infection in leaves from mock-treated (Control) and Sys, PS1-70 and PS1-120 treated plants. **(A)** Pictures show necrosis symptoms on detached tomato leaves 8 days after fungus inoculation. **(B)** The graphs display the mean of the lesion size at 1, 3, 5, and 8 days post-inoculum. Letters indicate different statistical groups (One-Way ANOVA, *P* < 0.05). Error bars indicate standard error.

Similarly, PS1-70 and PS1-120 were compared to Sys for the ability to control *B. cinerea* leaf colonization. Leaves treated with Sys, PS1-70 and PS1-120 showed a strong reduction of the leaf damages produced by the fungus already 1 day post-inoculum, and this difference increased over time, as shown in Figures 4A,B

In order to evaluate whether the reduction of *B. cinerea* necrosis area was due to a direct effect of fragments on the fungus, an *in vitro* assay was carried out. No impact of the tested fragments on fungus vitality was observed as shown in Supplementary Figure 6.

### Hydrogen Peroxide and Lipid Peroxidation Content in Tomato Leaves After ProSys Fragments Application

**FIGURE 5:**
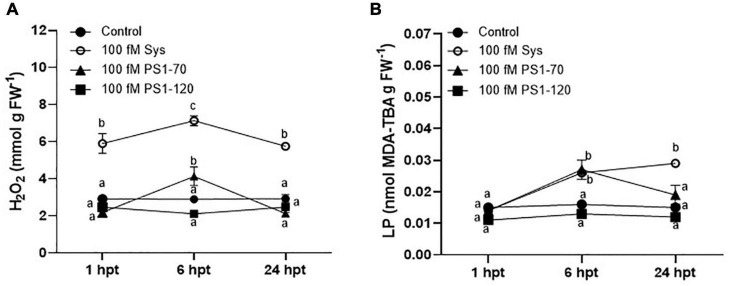
H_2_O_2_ and MDA content in tomato leaves treated with 100 fM ProSys fragments. H_2_O_2_. **(A)** and MDA, **(B)** contents were measured in control (PBS1X) and in treated leaves at 1, 6, and 24 hpt with 100 fM Sys, PS1-70 and PS1-120. Different letters indicate statistically significant differences (One-Way ANOVA, *P* < 0.05). Error bars indicate standard error.

We further investigated the effect of Sys, PS1-70 and PS1-120 fragments treatments on oxidative status in tomato plants after 1, 6, and 24 hours post treatment (hpt). As observed in Figure 5A, H_2_O_2_ content in Sys treated leaves was significantly higher than in untreated control at every time point, reaching its maximum level after 6 hpt [Tukey test, *F*(3.23) = 63.36; *P* < 0.0001]. Conversely, a different profile was observed for H_2_O_2_ content in PS1-70 and PS1-120 treated leaves. The former produced a significant increase of the ROS species only 6 hpt, while the latter did not affect H_2_O_2_ content. Coherently with these observed values, lipid peroxidation, determined by MDA content, showed no differences between PS1-120 treated plants and control, while a significant increase of MDA was observed in Sys and PS1-70 treated plants 6 hpt compared to controls (Figure 5B). In addition, in Sys treated leaves the lipid peroxidation showed a further increase at 24 hpt (Figure 5B).

### Induction of the Expression of Plant Defense-Related Genes After ProSys Fragments Application

The evaluation of the perception and the effect of the exogenous supply of Sys, PS1-70 and PS1-120 on the expression of defense genes was performed quantifying the transcripts of selected defense-related genes (listed in Supplementary Table 2) by qPCR, 6 h after peptide delivery on healthy leaves *Allene Oxide Synthase* (*AOS;* Solyc11g069800), *Lipoxygenase C* (*LoxC;* Solyc01g006540), *Lipoxygenase D* (*LoxD;* Solyc03g122340), *1-aminocyclopropane-1-carboxylate oxidase (ACO2;* Solyc12g005940), *Wound-induced Proteinase Inhinbitor I* and *II*, (*Pin I* and *Pin II;* Solyc09g084470 and Solyc03g020060), *Phenylalanine ammonia-lyase (PAL;* Solyc09g007910) were considered for the expression analyses.

**FIGURE 6:**
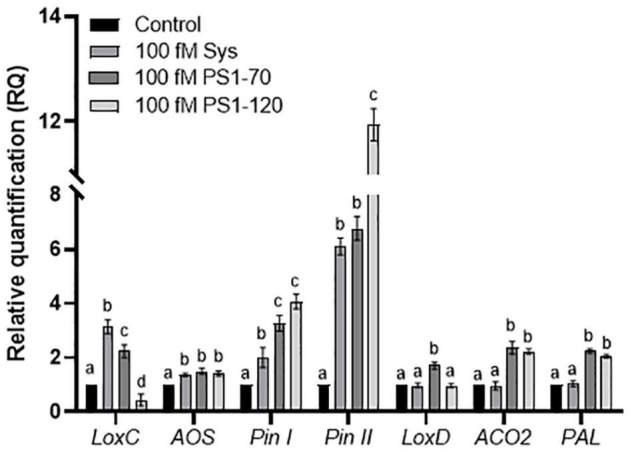
Expression analysis of defense related genes by RT-PCR. Relative quantification of early and late defense genes induced at 6 h post treatment with 100 fM of Sys, PS1-70 and PS1-120. Quantities are shown relative to the calibrator control condition, set as 1 (mock-treated plants). Letters indicate different statistical groups (One-Way ANOVA, *P* < 0.05). Error bars indicate standard error.

All the experimental peptides induced the expression of the selected genes as shown in Figure 6. Interestingly, some remarkable differences were observed in the level of transcripts encoding LoxC, Pin I and Pin II proteins. However, the expression of different genes was differently induced by the experimental peptides. AOS is similarly induced by the three peptides, LoxD transcription appears to be induced uniquely by PS1-70, while the transcription of ACO2 and PAL by PS1-70 and PS1-120, but not by Sys peptide.

### Effects of ProSys Fragments on Microorganism and Mammalian Cells

To evaluate if ProSys fragments under investigation have a cytotoxic effect on photosynthetic microorganism and human cells, we investigated cell viability and growth of fragment-treated microalgae and the proliferation and morphology of fragment-treated human keratinocytes (HaCaT).

Exposure of the microalgal cells to different concentration of ProSys fragments did not affect their viability. Growth curves of *Chlamydomonas* cells exposed at different concentrations of Sys, PS1-70 and PS1-120 were comparable to that of control cells, across the whole duration of the experiment (Supplementary Figure 7). Similarly, all experimental treatments did not inhibit growth of algae (Supplementary Table 5). Therefore, these results indicate that neither Sys nor the N-terminal fragments 1-70 and 1-120 of ProSys have toxicity effect on microalgae. Finally, even using peptide concentrations much higher than those used for plant treatments, toxicity was not observed.

**FIGURE 7:**
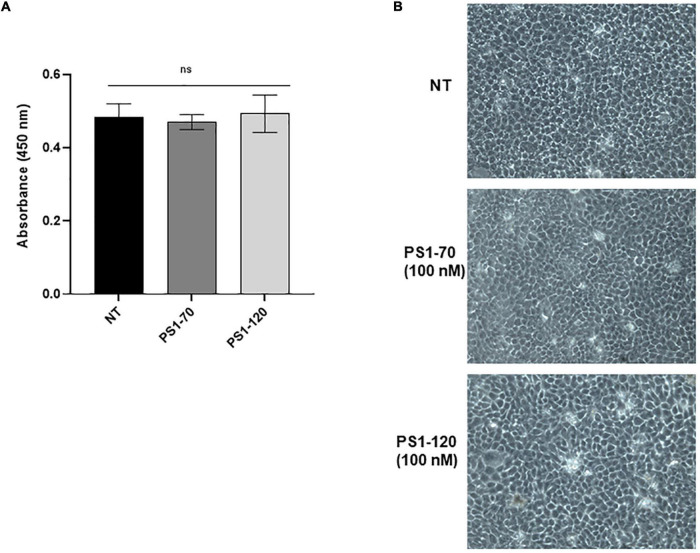
Cell viability and morphology of human keratinocyte cells (HaCaT cells) treated with PS1-70 or PS1-120. **(A)** Cell viability of HaCaT, incubated at 37°C for 24 h in the absence (NT) or presence of PS1-70 or PS1-120 at 100 nM, was evaluated by CCK-8 assay. **(B)** Phase-contrast microscopy images of HaCaT cells not treated (NT) or treated with 100 nM of PS1-70 or PS1-120 at 37°C for 24 h. The microscope images were representative of three independent experiments performed in triplicate. Bars are presented as mean (Student’s *t*-test, *P* < 0.05). Error bars indicate standard deviation.

The potential of PS1-70 or PS1-120 to affect mammalian cell proliferation and morphology was evaluated on human keratinocytes (HaCaT) cell line, exposing the cells at high concentrations (100 nM) of each peptide for 24 h. The cell line viability was assessed by cell viability Kit-8 assay and manual cell count, while the cell morphology was evaluated by phase-contrast microscopy. As reported in Figure 7A, following the treatment of HaCaT cells with 100 nM of each fragments for 24 h, the cell viability determined on the basis of absorbance of CCK-8 assay was about 100% (97.9% for PS1-70 and 100% for PS1-120), as also confirmed by the results obtained by manual cell count (94% for PS1-70 and 95% for PS1-120). These findings clearly revealed that PS1-70 and PS1-120 did not exert any cytotoxic effects against the mammalian cells used, even at the highest concentration tested (100 nM), which was much higher than that inducing the observed biological effects. In addition, to evidence possible alterations of cell morphology caused by treatments with ProSys fragments, HaCaT cell line was also visualized and analyzed by phase-contrast microscopy after 24 h exposure to 100 nM of PS1-70 or PS1-120. As shown in the micrographs reported in Figure 7B, both fragments did not induce any morphological change in the experimental cells.

## Discussion

The development of sustainable plant protection strategies is currently being pursued through a wealth of bioinspired approaches, based on the use of natural antagonists or of molecules/genes that modulate their interactions with target pests and pathogens, trying to control them by mimicking natural mechanisms of pest suppression (Pennacchio et al., 2012). Indeed, in addition to the use of living organisms, it is possible to use nature-based substances, such as, for example, plant-derived molecules (Isman, 2006), semiochemicals (Bruce et al., 2005; Witzgall et al., 2010), protein applications (Thakur and Sohal, 2013), and RNA interference (Zhu et al., 2011; Koch et al., 2016). The International Biocontrol Manufacturers’ Association (IBMA) promotes the broader term *bioprotection*, which includes the use of both biocontrol agents and non-living plant protection tools originated from nature (Stenberg et al., 2021). The growing knowledge on the functional basis of biological control allows to include in this definition also the use of molecules/genes deriving from natural antagonists, which can reproduce the lethal syndrome they induce in the target pests used as hosts (Bale et al., 2008; Pennacchio et al., 2012; Mahmood et al., 2016). Indeed, many bioactive molecules that regulate insect antagonistic interactions have already been isolated and characterized from plants and microorganisms (bacteria, fungi and viruses), to obtain insecticides of natural origins (Kachhawa, 2017), and many other untapped sources are available in nature.

This strategy can be applied to all kind of antagonistic associations existing in nature, including those among plants and their biotic stress agents. Natural molecules able to trigger plant immunity, such as DAMPs, may be interesting players in pest management, to activate defense responses; therefore, the discovery of novel DAMP-molecules may offer new opportunities for pest control, contributing to agro-food safety (Delaunois et al., 2014).

Here we describe the biological activity of two N-terminal fragments of ProSys, consisting of the first 70 and 120 amino acid residues, which, when exogenously supplied to tomato plants, protect them against *S. littoralis* larvae and *B. cinerea* infection.

SEC and LS investigations of PS1-70 and PS1-120, revealed the lack of a globular structure and a monomeric state in solution, in agreement with their disordered features, supported by CD and sequence analysis. As expected, the structural features of PS1-70 and PS1-120 strongly resemble those of the precursor (Radivojac et al., 2007; Midic and Obradovic, 2012; Buonanno et al., 2018; Uversky, 2019). When titrating with TFE, CD spectra showed that PS1-120 gained a more ordered three-dimensional structure compared with PS1-70, which retained most of its disordered conformation (Figures 2C,D). It is tempting to speculate that the conformational differences between the two fragments could promote a different recognition mechanism carried out by the same or by different partners (Uversky, 2019; Wang and Wang, 2019). However, this aspect deserves further investigations. In any case, the observed differences between PS1-70 and PS1-120, may well be dependent from their structures as PS1-70 encompasses the N-terminal region, which was predicted to be highly disordered, whereas PS1-120 also includes the central region 71-120, which was predicted to gain some secondary structure content.

Notably, PS1-70 and PS1-120, like Sys, possibly behave as hormone molecules being able to act at extremely low concentration, conferring to the treated plants different levels of protection against *S. littoralis* larvae and *B. cinerea*. The best performance of tomato plants against *S. littoralis* larvae was observed after the treatment with PS1-70, which induced 100% mortality 13 days after the treatment. Conversely, PS1-120 was more effective in reducing *B. cinerea* colonization, compared with Sys peptide and the PS1-70 fragment. The observed plant defense responses are mediated by PS1-70 and PS1-120 that, similarly to Sys (Coppola et al., 2019), likely penetrate the cell wall, by unknown mechanisms, and trigger the expression of defense-related genes. However, these protein fragments may contain shorter bioactive peptide sequences that are worth further research efforts.

The absence of direct effects of the investigated fragments on *S. littoralis* larvae and *B. cinerea* further demonstrates that plant protection is due to the induction of endogenous defenses, which is coupled with a high level of safety toward the non-target organisms considered.

We propose that both PS1-70 and PS1-120 can act as resistance inducer molecules, that enhance plant defense barriers upon challenge (Mauch-Mani et al., 2017). Indeed, PS1-70 and PS1-120 induce the expression of genes associated with the octadecanoid signaling pathway (*Lox C*, and *AOS*), that is known to play an important role in plant defense against a variety of insects and fungal pathogens (Thakur and Udayashankar, 2019; Viswanath et al., 2020; Zeng et al., 2021), and of jasmonic acid (JA) responsive genes (*Pin I* and *Pin II*), known to reduce larval nutrient digestion and, consequently, their growth and survival, as well as *B. cinerea* development, both *in vitro* and *in vivo* (Lorito et al., 1994; Hermosa et al., 2006). Intriguingly, PS1-70 induces the expression of *LoxD*, a gene that, when overexpressed in tomato plants, boosts JA biosynthesis, increasing the expression of wound-responsive genes and, consequently, enhancing resistance against insect herbivores (Yan et al., 2013). These observations nicely correlate with the efficient protection against the herbivorous pest triggered by PS1-70. In addition, PS1-70 and PS1-120 induced the expression of *ACO*, a gene associated with ethylene production (Houben and Van de Poel, 2019), an important regulator of plant responses to biotic and abiotic stress agents (Van de Poel et al., 2016), and of *Pal*, a gene encoding a key-enzyme of plant metabolism leading to changes in lignin contents and thickness of cell walls, involved in plant defense responses against parasites (Tonnessen et al., 2015; Chen et al., 2017).

Upon stress conditions, homeostasis of intracellular oxidation-reduction is impaired, with the consequent increase of ROS that damage the cells through lipid peroxidation (Huang et al., 2019). By measuring the malondialdehyde (MDA), which is the result of lipid peroxidation, the amount of plant cell stress can be measured (Grotto et al., 2009). Oxidative parameters showed an additional difference in terms of ROS generation among PS1-70, PS1-120 and Sys. Taken together, these results indicate that PS1-70, PS1-120 and Sys all confer protection to treated plants, but to a different extent, which partly accounts for the diversity of functional responses observed.

Importantly, Sys, PS1-70 and PS1-120 did not show toxic activity on microalgae and on HaCaT cells, making them suitable for crop protection.

Collectively, these results indicate that ProSys fragments act as multiple signals which may interact with different receptors controlling complementary and synergistic defense pathways (Zhang et al., 2020). This appears to be a widespread strategy of response to stress agents, which is able to rapidly produce multiple signals alerting the whole organism, to allow a more effective and rapid response against stress (Olsson et al., 2019; Stührwohldt and Schaller, 2019). The Hydroxyproline-rich systemins (HypSys) were the first example of production in plants of multiple bioactive peptides from a single precursor protein (Pearce et al., 2001; Pearce and Ryan, 2003). A similar gene, *SlpreproHypSys* was also described in tomato; the gene encodes a polypeptide of 145 amino acids from which three different small defense signaling glycopeptides are released (Ryan and Pearce, 2003). Several other examples were lately reported (Chen et al., 2020), evidencing the very important biological role played by plant peptides and protein fragments in physiological responses, which have been underestimated for many years.

Although ProSys was discovered several decades ago (McGurl et al., 1992), and many observations proved its important role in tomato protection, our results show for the first time that this protein contains multiple fragments with biological activity, likely accounting for the different “anti stress” activities conferred to tomato plants by its overexpression. The two ProSys fragments identified in this study represent new promising tools for sustainable crop protection. Any shorter bioactive sequence deriving from them is currently being investigated by us, to identify new plant protection products and biostimulants that will contribute to the important goal of reducing the use of synthetic pesticides in agriculture.

## Data Availability Statement

The raw data supporting the conclusions of this article will be made available by the authors, without undue reservation.

## Author Contributions

DM produced and structurally characterized ProSys fragments, performed the bioassays, and contributed to manuscript writing. MC participated to the experimental work and contributed to the manuscript writing. EL conceived the fragments and contributed to the manuscript writing. MB characterized the ProSys fragment and revised the manuscript. IDL, EB, and AB carried out the insect bioassays. AA performed the gene expression studies and insect and fungi assays. SMM supervised the biochemical work, conceived the work, and contributed to the manuscript writing. MR and SF performed the biochemical assays and manuscript revisions. PC carried out the microalgal assay. GP and RT performed the human cell assays. MS carried out the fungi bioassays. FP contributed to the insect work, supervision, and manuscript revision. RR conceived and supervised the work and wrote the manuscript. All authors contributed to the article and approved the submitted version.

## Conflict of Interest

The authors declare that the research was conducted in the absence of any commercial or financial relationships that could be construed as a potential conflict of interest.

## Publisher’s Note

All claims expressed in this article are solely those of the authors and do not necessarily represent those of their affiliated organizations, or those of the publisher, the editors and the reviewers. Any product that may be evaluated in this article, or claim that may be made by its manufacturer, is not guaranteed or endorsed by the publisher.

## Footnotes

1
https://www.fao.org/plant-health-2020/about/en/

2
http://dichroweb.cryst.bbk.ac.uk/html/home.shtml

3
http://www.cprofiler.org/

4
http://us.expasy.org/sprot
