# Supplementary material for: Not Only Systemin: Prosystemin Harbors Other Active Regions Able to Protect Tomato Plants
Source: Front Plant Sci. 2022 May 24;13:887674. doi: 10.3389/fpls.2022.887674 (PMC9173717; doi:10.3389/fpls.2022.887674)
Supplement: Supplementary file 1 [file Data_Sheet_1.pdf]

Supplementary Material

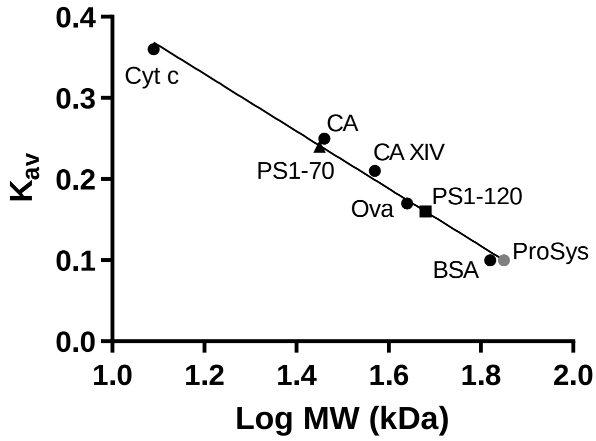

Supplementary Figure S1. Apparent molecular mass of PS1-70 and PS1-120. Apparent molecular mass of PS1-70 and PS1-120 as calculated by SEC. Calibration curve was built using five standard proteins (black circle). $K_{av} = (V_e\text{-}V_o/V_t\text{-}V_o)$, where V_e = protein elution volume, V_t = column volume, and V_o = void column volume. ProSys: grey circle; PS1-70: black triangle and PS1-120: black square.

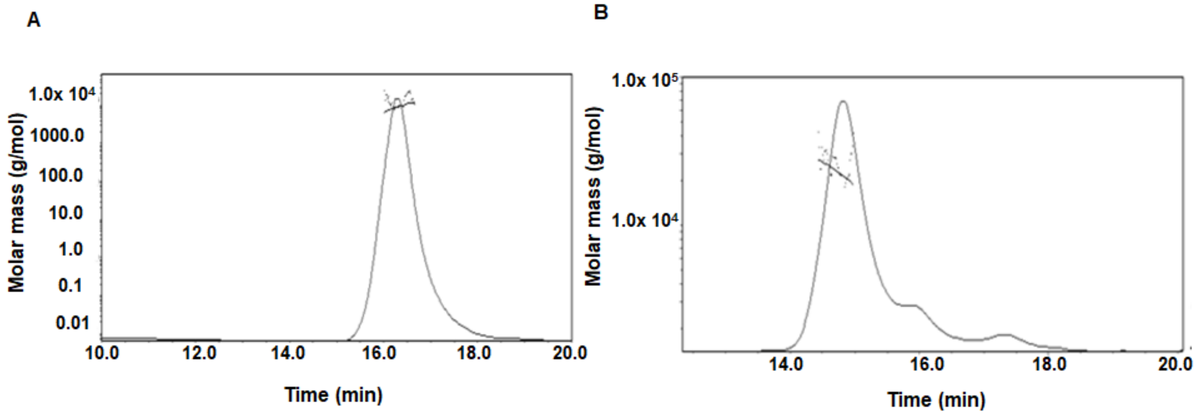

Supplementary Figure S2. Multi Angle Light Scattering of ProSys fragments. The peaks of **(A)** PS1-70 and **(B)** PS1-120 are representative of monomeric proteins.

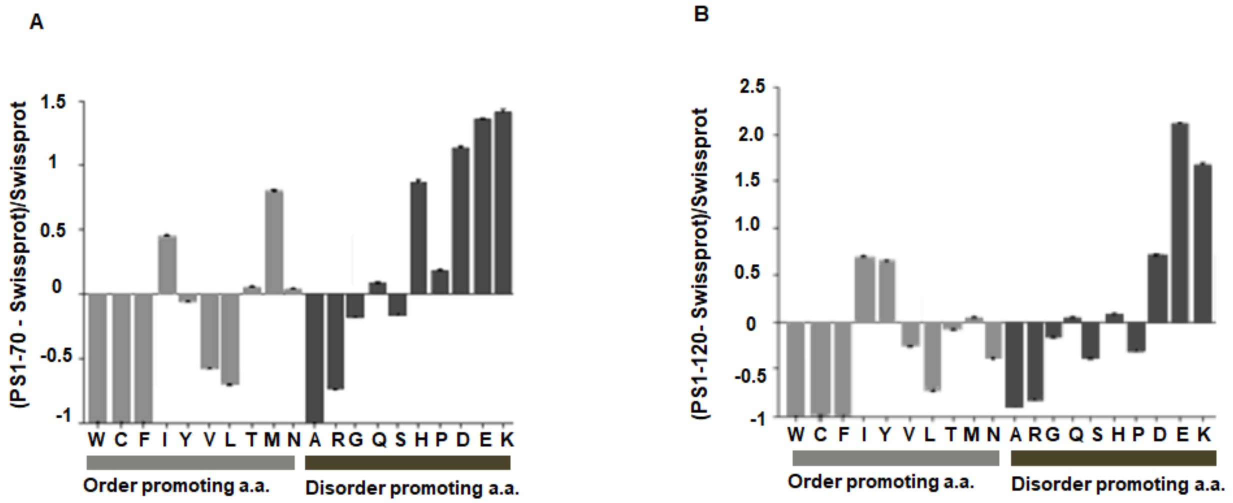

Supplementary Figure S3. Sequence properties of PS1-70 and PS1-120. Amino acid compositional analysis of **(A)** PS 1-70 and **(B)** PS 1-120 performed by Composition Profiler tool.

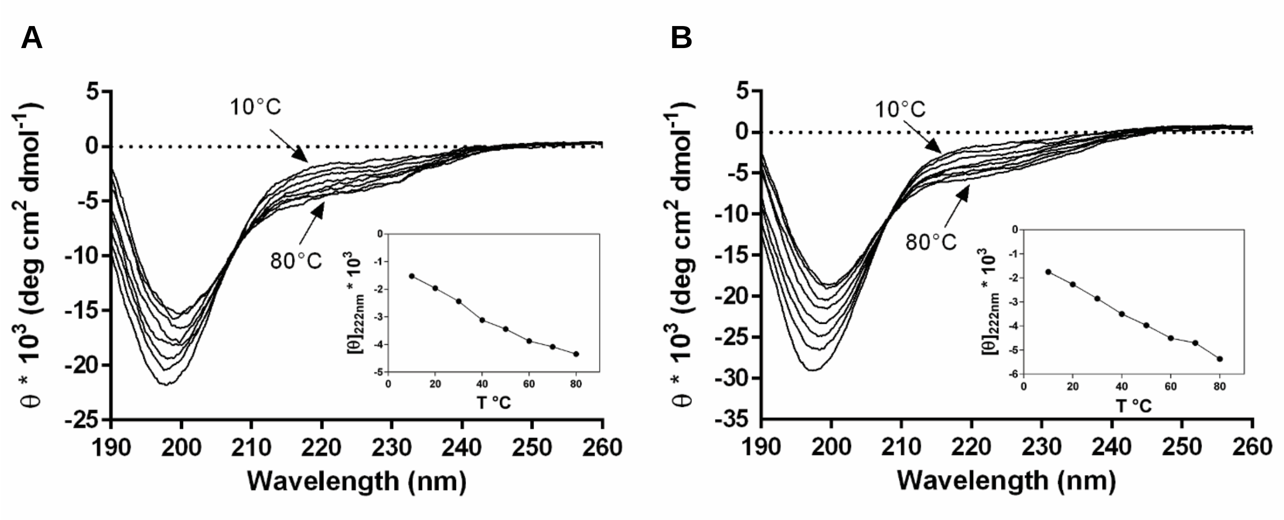

Supplementary Figure S4. CD spectra of PS1-70 and PS1-120 at different temperatures. Temperature effect on the secondary structure content of **(A)** PS1-70 and **(B)** PS1-120 at increasing temperature (10°C, 20°C, 30°C, 40°C, 50°C, 60°C, 70°C and 80°C). Changes in molar ellipticity at 222 nm as a function of the thermal treatment are shown in the inset for both fragments. Spectra were acquired in 10 mM phosphate buffer, pH 7.4.

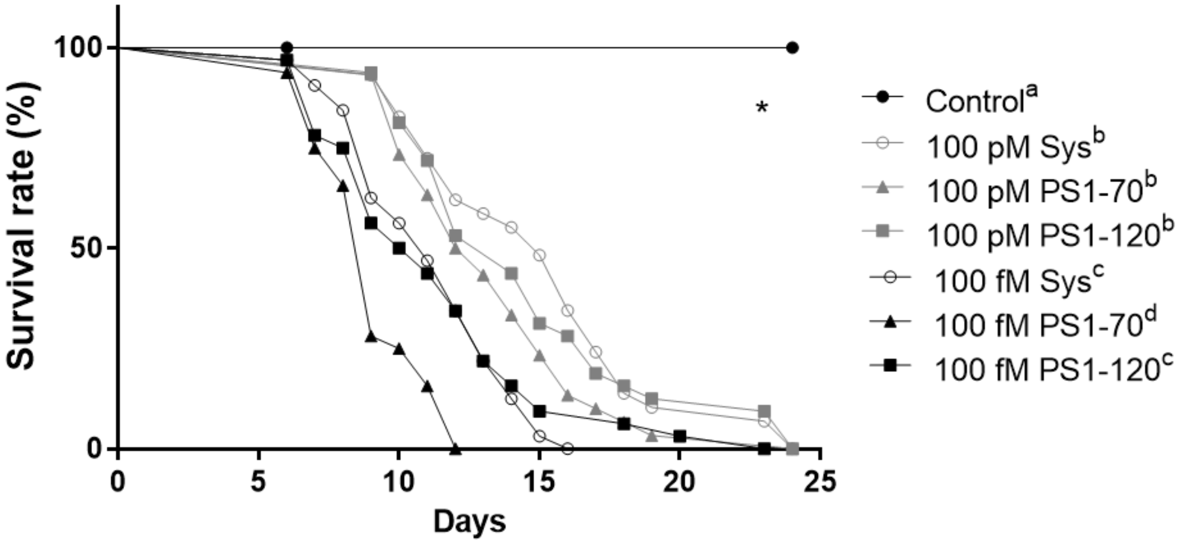

Supplementary Figure S5. Effect of 100 pM and 100 fM Sys, PS1-70 and PS1-120 on *S. littoralis* larvae. Survival rate of experimental *S. littoralis* larvae. Different letters denote significant differences in the survival curve (Log-Rank test, $P < 0.0001$).

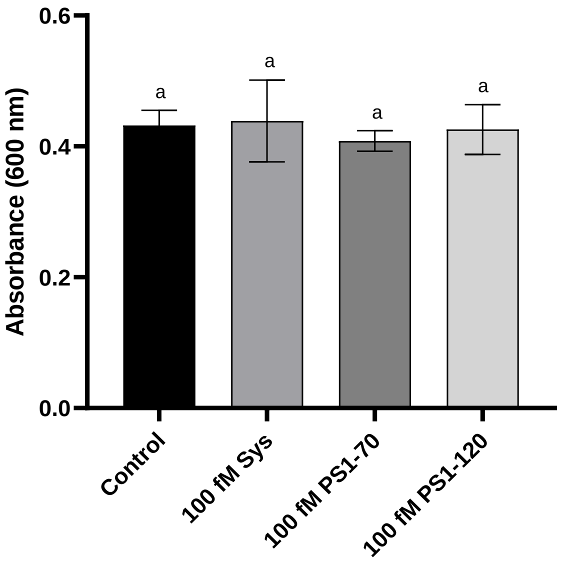

Supplementary Figure S6. *In vitro* antifungal vitality assay in presence of ProSys fragments. Spores of *B. cinerea* were added to each well filled with 1 ml of PDB1/2 medium containing the peptides at the final concentration of 100 fM, except for the broth sterility control wells. Fungal growth was assessed 24 hours after pathogen inoculation by evaluating the OD_{600} of the medium. Different letters indicate statistically significant differences (One-Way ANOVA, $P < 0.05$). Error bars indicate standard error.

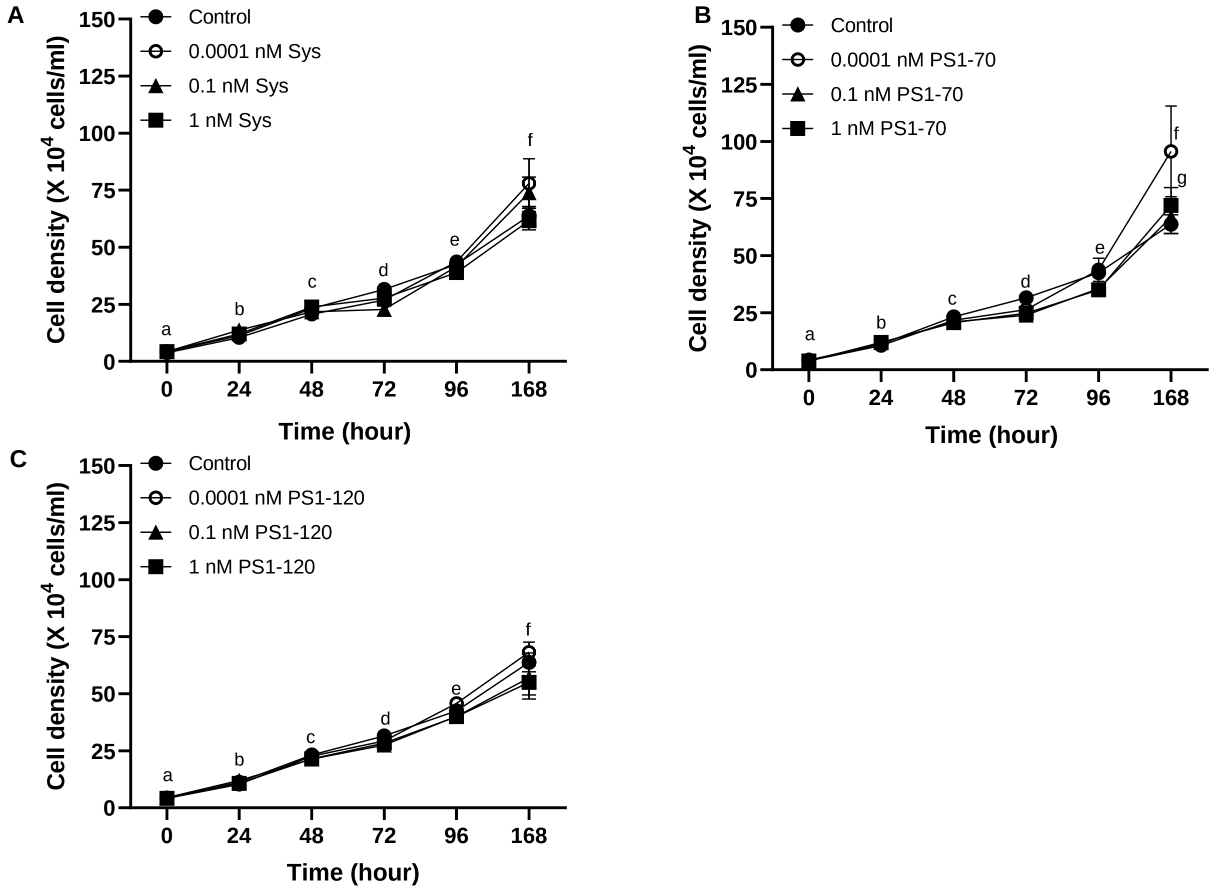

Supplementary Figure S7. Growth curves of *Chlamydomonas reinhardtii* treated with different concentrations of peptides. **(A)** Sys, **(B)** PS1-70, and **(C)** PS1-120, used at different concentrations. (One way ANOVA, $P < 0.05$, not significant). Error bars indicate standard deviation.

Supplementary Table S1. List of primer sequences, expected amplicon size and number of PCR cycles used.

Primer name	Sequence (5'-3')*	Fragment name	Amplified length (bp)	PCR cycle	Number of PCR cycle
**P11F1**<br>**P11R1**	CGCGCGCCATGGGAACTCCTTCATATGATATCA<br>CGCGCG*CTCGAG*TTACTACTCCTCATGTTCCATCTTTGGTA	PS1-70	236 bp	98°C for 10 seconds<br>66°C for 15 seconds<br>72°C for 30 seconds	30
**P11F1**<br>**P11R3**	CGCGCGCCATGGGAACTCCTTCATATGATATCA<br>CGCGCG*CTCGAG*TTACTAAGTCTCTTTTTCAACTATTTTCTCTTTTTC	PS1-120	386bp	98°C for 10 seconds<br>66°C for 15 seconds<br>72°C for 30 seconds	30

*NcoI restriction site is underlined; XhoI restriction site is italicized and underlined.

Supplementary Table S2. List of primers used for Real-time qPCR analysis.

Primer	Sequence (5'-3')	Amplicon lenght (bp)	Gene name	Accession number
ACO2 Fw	AACTCCTCAAAGACGGTGA	95	*ACO2*	Solyc12g005940
ACO2 Rv	TCCCGTTCGTGATTACCTCG			
AOS Fw	GATCGGTTCGTCGGAGAAGAA	101	*AOS*	Solyc11g069800
AOS Rv	GCGCACTGTTTATTCCCCACT			
EF Fw	CTCCATTGGGTCGTTTTGCT	101	*EF-1α*	Solyc06g005060
EF Rv	GGTCACCTTGGCACCAGTTG			
Lox C Fw	TTGCCTATGGTGCTGAATGGA	101	*Lox C*	Solyc01g006540
Lox C Rv	CAAGCCATGTGGTTCATTTGG			
Lox D Fw	TTCATGGCCGTGGTTGACA	102	*Lox D*	Solyc03g122340
Lox D Rv	AACAATCTCTGCATCTCCGG			
Pal Fw	CCACATTCAGCAACAAGGGC	126	*PAL*	Solyc09g007910
Pal Rv	ACACGGGGTAATGTTGCTGT			
Pin I Fw	GAAACTCTCATGGCACGAAAAG	114	*Pin I*	Solyc09g084470
Pin I Rv	CACCAATAAGTTCTGGCCACAT			
Pin II Fw	CCAAAAAGGCCAAATGCTTG	116	*Pin II*	Solyc03g020060
Pin II Rv	TGTGCAACACGTGGTACATCC			

Tm: melting temperature; LA: length amplicon; NC: number of cycles;[1] calculated according to the rule of Wallace: 4ºC for G and C, 2 °C for the A and T (Wallace, 1999).

Supplementary Table S3. Weight increase of *S. littoralis* larvae fed on leaves of tomato plants treated with Sys peptide, PS1-70 and PS1-120 fragments. Averages ± standard deviations are reported for Sys, PS1-70, PS1-120 and control (C) samples for all experimental time points. For each time point, mean values denoted with different letters are significantly different (One-way ANOVA: *P< 0.05; ** P< 0.0001 followed the Tukey-Kramer Honestly Significant Difference (HSD) multiple range test).

Days of feeding	C<br>Larval weight (g)	Systemin<br>Larval weight (g)	PS1-70<br>Larval weight (g)	PS1-120<br>Larval weight (g)	F	P value
1	**0.002** ± 3.33E-05 [a]	**0.0023** ± 2.94E-05 [a]	**0.0024** ± 3.41E-05 [a]	**0.0023** ± 3.24E-05 [a]	F (3. 124) = 0.5599	0.6425
3	**0.004** ± 7.98E-05 [a]	**0.0032** ± 7.22E-05 [b]	**0.0033** ± 9.64E-05 [b]	**0.0035** ± 0.0001 [b]	F (3. 123) = 15.27	< 0.0001**
5	**0.0159** ± 0.0036[a]	**0.0099** ± 0.0026 [ab]	**0.0068** ± 0.0004 [b]	**0.0070** ± 0.0004 [b]	F (3. 124) = 3.619	0.0151*
7	**0.0153** ± 0.0006 [a]	**0.0092** ± 0.0007 [b]	**0.0079** ± 0.0008 [b]	**0.0087** ± 0.0006 b	F (3. 107) = 27.86	< 0.0001**
9	**0.0240** ± 0.0006 [a]	**0.0082** ± 0.0007 [b]	**0.0084**± 0.0007 [b]	**0.0093** ± 0.0006 [b]	F (3. 75) = 147.4	< 0.0001**
11	**0.0346** ± 0.0007 [a]	**0.0118** ± 0.0010 [b]	**0.0117** ± 0.0011 [b]	**0.0135** ± 0.0013 [b]	F (3. 64) = 176.2	< 0.0001**
13	**0.0439** ± 0.0012 [a]	**0.0157** ± 0.0020 [b]	**0.0109** ± 0.0029 [b]	**0.0115** ± 0.0020 b	F (3. 44) = 92.95	< 0.0001**

Supplementary Table S4. Evaluation of larvicidal effect of the direct delivery of ProSys fragments at different concentrations on *Spodoptera littoralis* larvae.

**Oral ingestion**	**Control**	**100 fM Sys**	**100 pM Sys**	**100 nM Sys**	**100 fM PS1-70**	**100 fM PS1-70**	**100 nM PS1-70**	**100 fM PS1-120**	**100 pM PS1-120**	**100 nM PS1-120**
**4th instar**	16	16	16	16	16	16	16	16	16	16
**pupal stage**	15	16	15	16	16	15	15	16	16	15
**% survival**	93.75	100	93.75	100	100	93.75	93.75	100	100	93.75
**Topical application**	**Control**	**100 fM Sys**	**100 pM Sys**	**100 nM Sys**	**100 fM PS1-70**	**100 fM PS1-70**	**100 nM PS1-70**	**100 fM PS1-120**	**100 pM PS1-120**	**100 nM PS1-120**
**4th instar**	16	16	16	16	16	16	16	16	16	16
**pupal stage**	15	16	16	16	15	16	15	15	16	16
**% survival**	93.75	100	100	100	93.75	100	93.75	93.75	100	100

Supplementary Table S5. *C. reinhardtii* 96 hours growth rate inhibition using different concentration of Sys, PS1-70 and PS1-120.

Sample	Concentration (nM)	Growth rate inhibition (% of control)
Sys	0.0001	-7.9 ±1.8
	0.1	1,2 ±1.7
	1	3.4 ± 2.9
PS1-70	0.0001	-4.7 ± 3.9
	0.1	3 ± 2.6
	1	4.2 ± 2
PS1-120	0.0001	-4.7 ±0.9
	0.1	3.8 ± 1.3
	1	1.3 ± 1.3
